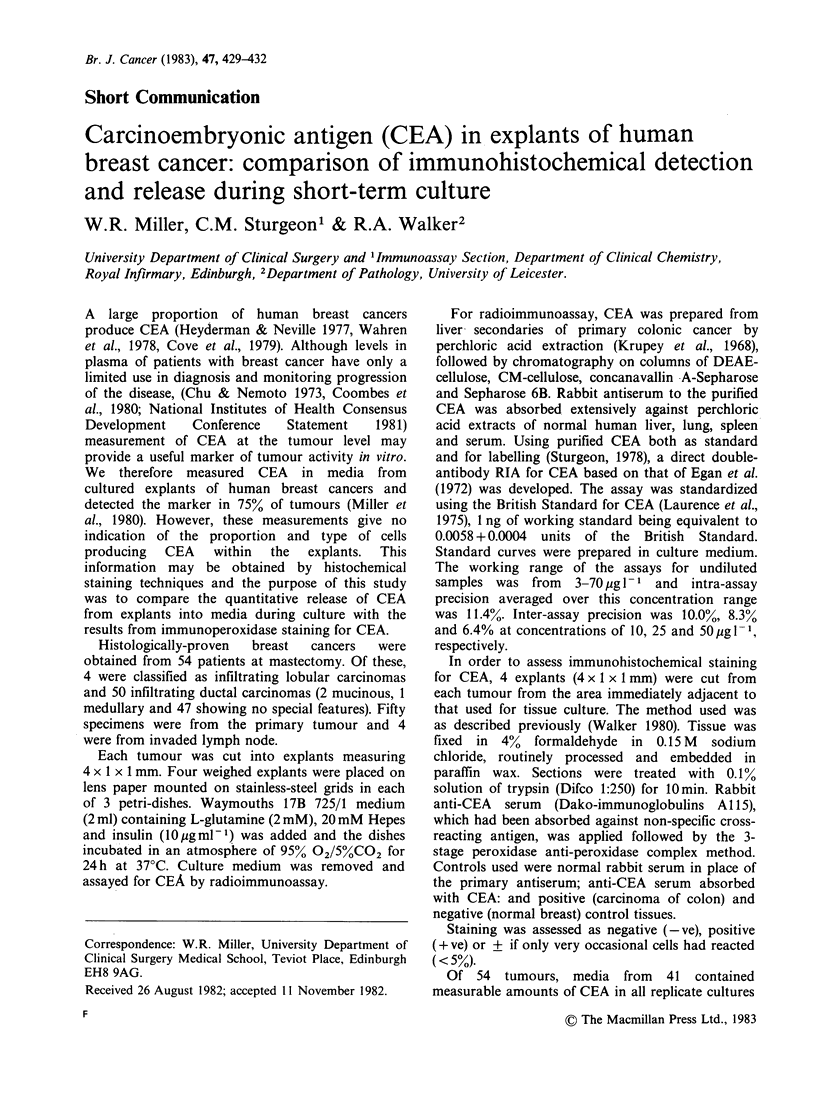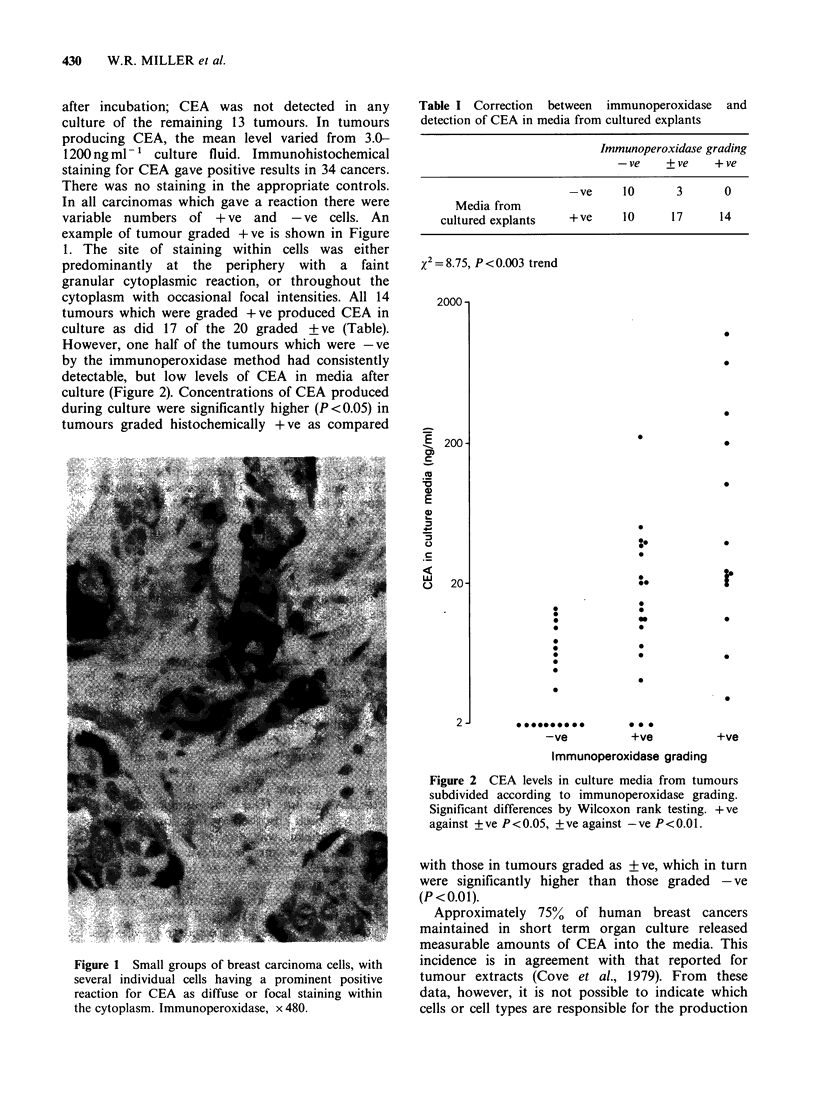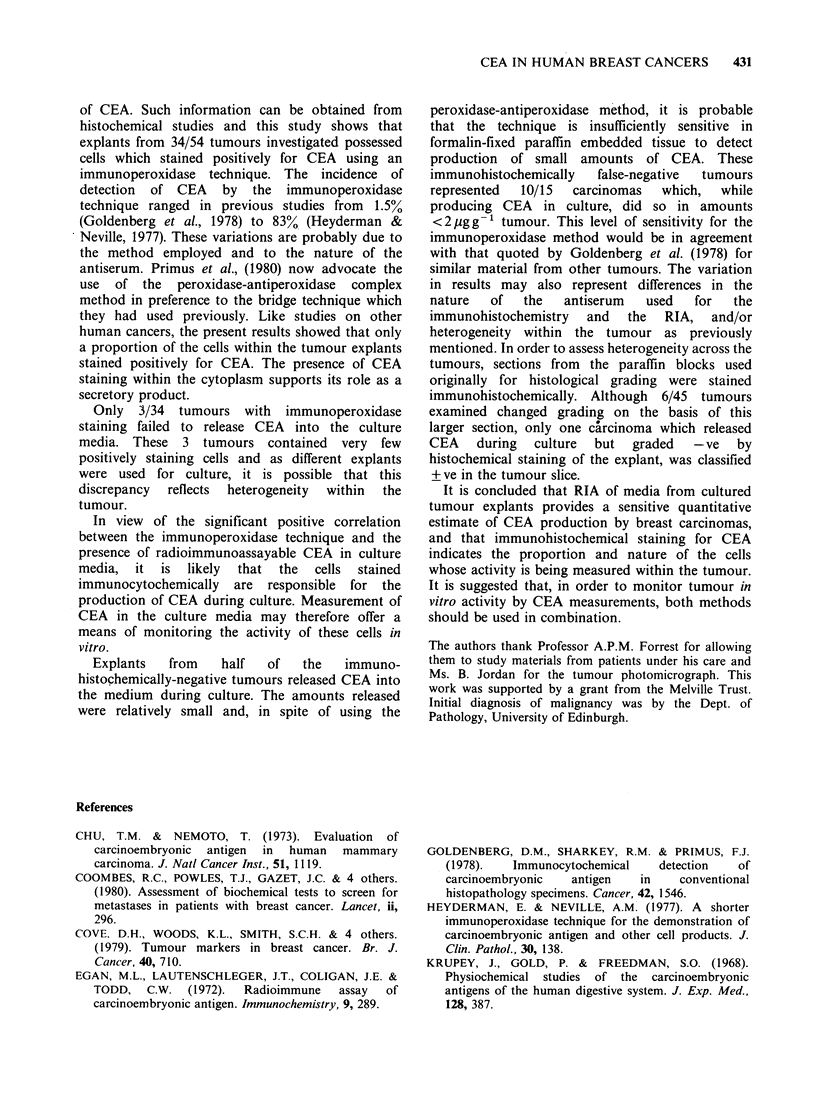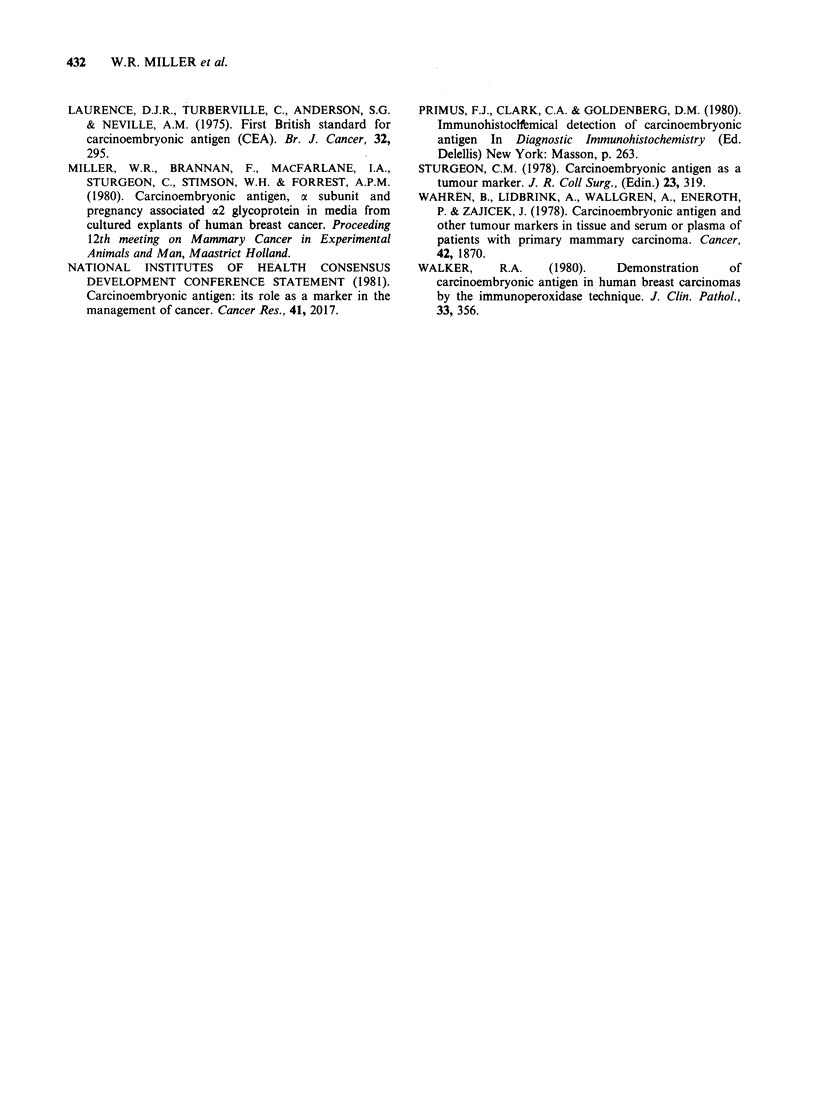# Carcinoembryonic antigen (CEA) in explants of human breast cancer: comparison of immunohistochemical detection and release during short-term culture.

**DOI:** 10.1038/bjc.1983.65

**Published:** 1983-03

**Authors:** W. R. Miller, C. M. Sturgeon, R. A. Walker

## Abstract

**Images:**


*Br. J. Cancer* (1983), **47**, 429–432

### Short Communication

### Carcinoembryonic antigen (CEA) in explants of human breast cancer: comparison of immunohistochemical detection and release during short-term culture

W.R. Miller, C.M. Sturgeon[1] & R.A. Walker[2]

*University Department of Clinical Surgery and [1]Immunoassay Section, Department of Clinical Chemistry, Royal Infirmary, Edinburgh, [2]Department of Pathology, University of Leicester.*

A large proportion of human breast cancers produce CEA (Heyderman & Neville 1977, Wahren *et al.*, 1978, Cove *et al.*, 1979). Although levels in plasma of patients with breast cancer have only a limited use in diagnosis and monitoring progression of the disease, (Chu & Nemoto 1973, Coombes *et al.*, 1980; National Institutes of Health Consensus Development Conference Statement 1981) measurement of CEA at the tumour level may provide a useful marker of tumour activity *in vitro*. We therefore measured CEA in media from cultured explants of human breast cancers and detected the marker in 75% of tumours (Miller *et al.*, 1980). However, these measurements give no indication of the proportion and type of cells producing CEA within the explants. This information may be obtained by histochemical staining techniques and the purpose of this study was to compare the quantitative release of CEA from explants into media during culture with the results from immunoperoxidase staining for CEA.

Histologically-proven breast cancers were obtained from 54 patients at mastectomy. Of these, 4 were classified as infiltrating lobular carcinomas and 50 infiltrating ductal carcinomas (2 mucinous, 1 medullary and 47 showing no special features). Fifty specimens were from the primary tumour and 4 were from invaded lymph node.

Each tumour was cut into explants measuring 4 x 1 x 1 mm. Four weighed explants were placed on lens paper mounted on stainless-steel grids in each of 3 petri-dishes. Waymouths 17B 725/1 medium (2 ml) containing L-glutamine (2 mM), 20 mM Hepes and insulin ($10\,\mu g\,ml^{-1}$) was added and the dishes incubated in an atmosphere of 95% $O_2$/5%$CO_2$ for 24 h at 37°C. Culture medium was removed and assayed for CEA by radioimmunoassay.

For radioimmunoassay, CEA was prepared from liver secondaries of primary colonic cancer by perchloric acid extraction (Krupey *et al.*, 1968), followed by chromatography on columns of DEAE-cellulose, CM-cellulose, concanavallin A-Sepharose and Sepharose 6B. Rabbit antiserum to the purified CEA was absorbed extensively against perchloric acid extracts of normal human liver, lung, spleen and serum. Using purified CEA both as standard and for labelling (Sturgeon, 1978), a direct double-antibody RIA for CEA based on that of Egan *et al.* (1972) was developed. The assay was standardized using the British Standard for CEA (Laurence *et al.*, 1975), 1 ng of working standard being equivalent to 0.0058 + 0.0004 units of the British Standard. Standard curves were prepared in culture medium. The working range of the assays for undiluted samples was from $3–70\,\mu g\,l^{-1}$ and intra-assay precision averaged over this concentration range was 11.4%. Inter-assay precision was 10.0%, 8.3% and 6.4% at concentrations of 10, 25 and $50\,\mu g\,l^{-1}$, respectively.

In order to assess immunohistochemical staining for CEA, 4 explants (4 x 1 x 1 mm) were cut from each tumour from the area immediately adjacent to that used for tissue culture. The method used was as described previously (Walker 1980). Tissue was fixed in 4% formaldehyde in 0.15 M sodium chloride, routinely processed and embedded in paraffin wax. Sections were treated with 0.1% solution of trypsin (Difco 1:250) for 10 min. Rabbit anti-CEA serum (Dako-immunoglobulins A115), which had been absorbed against non-specific cross-reacting antigen, was applied followed by the 3-stage peroxidase anti-peroxidase complex method. Controls used were normal rabbit serum in place of the primary antiserum; anti-CEA serum absorbed with CEA: and positive (carcinoma of colon) and negative (normal breast) control tissues.

Staining was assessed as negative (−ve), positive (+ve) or ± if only very occasional cells had reacted (<5%).

Of 54 tumours, media from 41 contained measurable amounts of CEA in all replicate cultures

Correspondence: W.R. Miller, University Department of Clinical Surgery Medical School, Teviot Place, Edinburgh EH8 9AG.

Received 26 August 1982; accepted 11 November 1982.

F

© The Macmillan Press Ltd., 1983

after incubation; CEA was not detected in any culture of the remaining 13 tumours. In tumours producing CEA, the mean level varied from 3.0–1200 ng ml$^{-1}$ culture fluid. Immunohistochemical staining for CEA gave positive results in 34 cancers. There was no staining in the appropriate controls. In all carcinomas which gave a reaction there were variable numbers of +ve and −ve cells. An example of tumour graded +ve is shown in Figure 1. The site of staining within cells was either predominantly at the periphery with a faint granular cytoplasmic reaction, or throughout the cytoplasm with occasional focal intensities. All 14 tumours which were graded +ve produced CEA in culture as did 17 of the 20 graded ±ve (Table). However, one half of the tumours which were −ve by the immunoperoxidase method had consistently detectable, but low levels of CEA in media after culture (Figure 2). Concentrations of CEA produced during culture were significantly higher ($P<0.05$) in tumours graded histochemically +ve as compared with those in tumours graded as ±ve, which in turn were significantly higher than those graded −ve ($P<0.01$).

**Figure 1** Small groups of breast carcinoma cells, with several individual cells having a prominent positive reaction for CEA as diffuse or focal staining within the cytoplasm. Immunoperoxidase, ×480.

**Table I** Correction between immunoperoxidase and detection of CEA in media from cultured explants

| | | *Immunoperoxidase grading* | | |
|---|---|---|---|---|
| | | *− ve* | *± ve* | *+ ve* |
| Media from cultured explants | − ve | 10 | 3 | 0 |
| | + ve | 10 | 17 | 14 |

$\chi^2 = 8.75$, $P<0.003$ trend

**Figure 2** CEA levels in culture media from tumours subdivided according to immunoperoxidase grading. Significant differences by Wilcoxon rank testing. +ve against ±ve $P<0.05$, ±ve against −ve $P<0.01$.

Approximately 75% of human breast cancers maintained in short term organ culture released measurable amounts of CEA into the media. This incidence is in agreement with that reported for tumour extracts (Cove *et al.*, 1979). From these data, however, it is not possible to indicate which cells or cell types are responsible for the production of CEA. Such information can be obtained from histochemical studies and this study shows that explants from 34/54 tumours investigated possessed cells which stained positively for CEA using an immunoperoxidase technique. The incidence of detection of CEA by the immunoperoxidase technique ranged in previous studies from 1.5% (Goldenberg *et al.*, 1978) to 83% (Heyderman & Neville, 1977). These variations are probably due to the method employed and to the nature of the antiserum. Primus *et al.*, (1980) now advocate the use of the peroxidase-antiperoxidase complex method in preference to the bridge technique which they had used previously. Like studies on other human cancers, the present results showed that only a proportion of the cells within the tumour explants stained positively for CEA. The presence of CEA staining within the cytoplasm supports its role as a secretory product.

Only 3/34 tumours with immunoperoxidase staining failed to release CEA into the culture media. These 3 tumours contained very few positively staining cells and as different explants were used for culture, it is possible that this discrepancy reflects heterogeneity within the tumour.

In view of the significant positive correlation between the immunoperoxidase technique and the presence of radioimmunoassayable CEA in culture media, it is likely that the cells stained immunocytochemically are responsible for the production of CEA during culture. Measurement of CEA in the culture media may therefore offer a means of monitoring the activity of these cells *in vitro*.

Explants from half of the immunohistochemically-negative tumours released CEA into the medium during culture. The amounts released were relatively small and, in spite of using the peroxidase-antiperoxidase method, it is probable that the technique is insufficiently sensitive in formalin-fixed paraffin embedded tissue to detect production of small amounts of CEA. These immunohistochemically false-negative tumours represented 10/15 carcinomas which, while producing CEA in culture, did so in amounts $<2\,\mu g\,g^{-1}$ tumour. This level of sensitivity for the immunoperoxidase method would be in agreement with that quoted by Goldenberg *et al.* (1978) for similar material from other tumours. The variation in results may also represent differences in the nature of the antiserum used for the immunohistochemistry and the RIA, and/or heterogeneity within the tumour as previously mentioned. In order to assess heterogeneity across the tumours, sections from the paraffin blocks used originally for histological grading were stained immunohistochemically. Although 6/45 tumours examined changed grading on the basis of this larger section, only one carcinoma which released CEA during culture but graded $-$ve by histochemical staining of the explant, was classified $\pm$ve in the tumour slice.

It is concluded that RIA of media from cultured tumour explants provides a sensitive quantitative estimate of CEA production by breast carcinomas, and that immunohistochemical staining for CEA indicates the proportion and nature of the cells whose activity is being measured within the tumour. It is suggested that, in order to monitor tumour *in vitro* activity by CEA measurements, both methods should be used in combination.

The authors thank Professor A.P.M. Forrest for allowing them to study materials from patients under his care and Ms. B. Jordan for the tumour photomicrograph. This work was supported by a grant from the Melville Trust. Initial diagnosis of malignancy was by the Dept. of Pathology, University of Edinburgh.

### References

CHU, T.M. & NEMOTO, T. (1973). Evaluation of carcinoembryonic antigen in human mammary carcinoma. *J. Natl Cancer Inst.*, **51**, 1119.

COOMBES, R.C., POWLES, T.J., GAZET, J.C. & 4 others. (1980). Assessment of biochemical tests to screen for metastases in patients with breast cancer. *Lancet*, **ii**, 296.

COVE, D.H., WOODS, K.L., SMITH, S.C.H. & 4 others. (1979). Tumour markers in breast cancer. *Br. J. Cancer*, **40**, 710.

EGAN, M.L., LAUTENSCHLEGER, J.T., COLIGAN, J.E. & TODD, C.W. (1972). Radioimmune assay of carcinoembryonic antigen. *Immunochemistry*, **9**, 289.

GOLDENBERG, D.M., SHARKEY, R.M. & PRIMUS, F.J. (1978). Immunocytochemical detection of carcinoembryonic antigen in conventional histopathology specimens. *Cancer*, **42**, 1546.

HEYDERMAN, E. & NEVILLE, A.M. (1977). A shorter immunoperoxidase technique for the demonstration of carcinoembryonic antigen and other cell products. *J. Clin. Pathol.*, **30**, 138.

KRUPEY, J., GOLD, P. & FREEDMAN, S.O. (1968). Physiochemical studies of the carcinoembryonic antigens of the human digestive system. *J. Exp. Med.*, **128**, 387.

LAURENCE, D.J.R., TURBERVILLE, C., ANDERSON, S.G. & NEVILLE, A.M. (1975). First British standard for carcinoembryonic antigen (CEA). *Br. J. Cancer,* **32,** 295.

MILLER, W.R., BRANNAN, F., MACFARLANE, I.A., STURGEON, C., STIMSON, W.H. & FORREST, A.P.M. (1980). Carcinoembryonic antigen, α subunit and pregnancy associated α2 glycoprotein in media from cultured explants of human breast cancer. *Proceeding 12th meeting on Mammary Cancer in Experimental Animals and Man, Maastrict Holland.*

NATIONAL INSTITUTES OF HEALTH CONSENSUS DEVELOPMENT CONFERENCE STATEMENT (1981). Carcinoembryonic antigen: its role as a marker in the management of cancer. *Cancer Res.,* **41,** 2017.

PRIMUS, F.J., CLARK, C.A. & GOLDENBERG, D.M. (1980). Immunohistochemical detection of carcinoembryonic antigen In *Diagnostic Immunohistochemistry* (Ed. Delellis) New York: Masson, p. 263.

STURGEON, C.M. (1978). Carcinoembryonic antigen as a tumour marker. *J. R. Coll Surg.,* (Edin.) **23,** 319.

WAHREN, B., LIDBRINK, A., WALLGREN, A., ENEROTH, P. & ZAJICEK, J. (1978). Carcinoembryonic antigen and other tumour markers in tissue and serum or plasma of patients with primary mammary carcinoma. *Cancer,* **42,** 1870.

WALKER, R.A. (1980). Demonstration of carcinoembryonic antigen in human breast carcinomas by the immunoperoxidase technique. *J. Clin. Pathol.,* **33,** 356.